# Antibacterial activity and mode of action of potassium tetraborate tetrahydrate against soft-rot bacterial plant pathogens

**DOI:** 10.1099/mic.0.000948

**Published:** 2020-07-08

**Authors:** Yingyu Liu, Melanie J. Filiatrault

**Affiliations:** ^1^​ Section of Plant Pathology and Plant-Microbe Biology, School of Integrative Plant Science, Cornell University, Ithaca, NY 14853, USA; ^2^​ Emerging Pests and Pathogens Research Unit, Robert W. Holley Center for Agriculture and Health, Agricultural Research Service, United States Department of Agriculture, Ithaca, NY 14853, USA

**Keywords:** *Dickeya*, *Pectobacterium*, potassium tetraborate tetrahydrate, chemical resistance, peptide chain release factor

## Abstract

Bacterial soft rot caused by the bacteria *Dickeya* and *Pectobacterium* is a destructive disease of vegetables, as well as ornamental plants. Several management options exist to help control these pathogens. Because of the limited success of these approaches, there is a need for the development of alternative methods to reduce losses. In this study, we evaluated the effect of potassium tetraborate tetrahydrate (PTB) on the growth of six *Dickeya* and *Pectobacterium* spp. Disc diffusion assays showed that *Dickeya* spp. and *Pectobacterium* spp. differ in their sensitivity to PTB. Spontaneous PTB-resistant mutants of *Pectobacterium* were identified and further investigation of the mechanism of PTB resistance was conducted by full genome sequencing. Point mutations in genes *cpdB* and *supK* were found in a single *Pectobacterium atrosepticum* PTB-resistant mutant. Additionally, point mutations in genes *prfB* (synonym *supK*) and *prmC* were found in two independent *Pectobacterium brasiliense* PTB-resistant mutants. *prfB* and *prmC* encode peptide chain release factor 2 and its methyltransferase, respectively. We propose the disruption of translation activity due to PTB leads to *Pectobacterium* growth inhibition. The *P. atrosepticum* PTB-resistant mutant showed altered swimming motility. Disease severity was reduced for *P. atrosepticum *-inoculated potato stems sprayed with PTB. We discuss the potential risk of selecting for bacterial resistance to this chemical.

## Data Summary

The raw Illumina sequencing reads of the following strains have been deposited in the National Center for Biotechnology Information (NCBI) Sequence Read Archive (SRA): *Pectobacterium atrosepticum* SCRI1043 wild-type (SRR10210572), *P. atrosepticum* SCRI1043 potassium tetraborate tetrahydrate (PTB)-resistant mutant (SRR10210571), *Pectobacterium brasiliense* 1692 wild-type (SRR10850514), *P. brasiliense* 1692 PTB-resistant mutant A (SRR11028658) and *P. brasiliense* 1692 PTB-resistant mutant B (SRR11028671). The complete genome sequence of *P. brasiliense* 1692 has been deposited at NCBI GenBank under the accession number CP047495.

## Introduction

Quantitative losses due to plant diseases caused by *Dickeya* and *Pectobacterium* spp. are greater than the losses caused by other bacterial plant pathogens [1]. *Dickeya* and *Pectobacterium* spp. are Gram-negative pectolytic bacteria that are ubiquitously present on the surface of potato seed pieces, in the soil [2], in surface water and in ground water [3, 4]. Potato diseases, including blackleg and tuber soft rot, can cause up to 60 % yield losses in the field, in transit and during storage [5]. Potato blackleg and soft-rot diseases are mainly controlled using cultural practices, while suitable and effective chemical treatment options are being explored. Current disinfecting agents used for potato storage include hypochlorites, chlorine dioxide, copper quinolinolate, quaternary ammonium and hydrogen peroxide [6]. Some inorganic salts, such as aluminium chloride, sodium benzoate and sodium thiosulfate, have been shown to inhibit the growth of *Pectobacterium* spp. *in vitro*, as well as on potato tubers [7]. The capacity of bacterial pathogens to develop resistance to chemicals and concerns about the negative impacts of chemicals on human health have promoted exploration of alternative environmentally safe bacteriocides.

Recently, an inorganic salt, potassium tetraborate tetrahydrate (PTB), was shown to alleviate soft-rot disease caused by *Pectobacterium carotovorum* BA17 on tomato fruits [8]. Several studies have demonstrated the potency of PTB in fungal pathosystems, such as grey mould of grapevines and tomato [9, 10], decay of melons [11, 12], citrus green mould [13] and mango anthracnose [14, 15]. Although PTB has been used in agriculture as a low impact chemical to treat *Penicillium* spp. and *Botrytis* spp. [16, 17], it has not been widely applied to control bacteria causing potato diseases. Several potential mechanisms for the inhibitory effect of PTB on pathogens have been described. Yaganza *et al*. proposed that elevated osmolality caused by inorganic salts may trigger increased maintenance metabolism and, therefore, inhibit bacterial reproduction [7]. Ahmed *et al*. suggested that the pH conditions of the PTB solution disrupt cell integrity and, therefore, have an adverse effect on the bacterial population [8]. The molecular mode of action of PTB against bacterial plant pathogens has not been demonstrated and, thus, remains unclear.

The objective of the current study was to determine the effect of PTB in inhibiting the growth of *Dickeya* and *Pectobacterium* spp., and to unravel the mode of action of PTB. We found that PTB was effective in inhibiting the growth of *Pectobacterium atrosepticum* SCRI1043, *P. carotovorum* WPP14, *Pectobacterium brasiliense* 1692 and *Pectobacterium parmentieri* WPP163 *in vitro*, but was less effective at inhibiting the growth of *Dickeya dadantii* 3937 and *Dickeya dianthicola* ME23. PTB-resistant mutant strains and point mutations were identified in *P. atrosepticum* and *P. brasiliense *. The efficacy of spraying PTB onto potato plants inoculated with *P. atrosepticum* was evaluated. The use of PTB as an inexpensive antibacterial chemical agent for controlling soft-rot pathogens is discussed.

## Methods

### Bacterial strains

*D. dadantii* 3937, *P. atrosepticum* SCRI1043, *P. carotovorum* WPP14, *P. carotovorum* subsp. *brasiliense* 1692, *P. parmentieri* WPP163 and *D. dianthicola* ME23 were regularly cultured at 28 °C on lysogeny broth (LB) agar plates or in LB (per 1 l medium: 10 g tryptone, 5 g yeast extract, 10 g NaCl, with 16 g agar for plates) [18].

### Antibacterial activity of PTB

The disc diffusion method was used to evaluate the antimicrobial activity of PTB on *Dickeya* and *Pectobacterium* spp. A total of 100 µl bacterial suspension of 2×10^8^ c.f.u. ml^−1^ (optical density at 600 nm wavelength ≈ 0.2) was spread onto individual LB agar plates. The plates were allowed to dry for 5 min under aseptic conditions. A 500 mM PTB solution was sterilized through a Millipore filter (0.22 µm) and 20 µl PTB solution was loaded onto each sterile filter paper disc (6 mm in diameter; GE Healthcare). Each disc with PTB was placed onto the centre of an agar plate that had been spread with bacterial suspension. Plates were incubated at 28 °C. After 24 h, pictures and diameter measurements were taken. The assay was performed in triplicate and repeated three times. A two-tailed Student's *t*-test (unequal variances) was used to check the statistical significance of differences between treatments (*P* value <0.05). The vertical lines in the graphs represent the standard deviation of the mean.

### Growth curve

Bacterial suspensions were adjusted to OD_600_ ≈ 0.1 in LB liquid medium and 200 µl of the bacterial suspensions with different concentrations of PTB were aliquoted into wells on a 96-well plate. The bacterial growth curve was created by monitoring the OD_600_ using a Synergy 2 multi-mode microplate reader (BioTek) for up to 12 h at 30 min intervals. At least six technical replicates per treatment and three biological replicates were performed. The vertical lines in the graphs represent the standard deviation of the mean.

### Whole-genome sequencing of spontaneous PTB-resistant mutants

Spontaneous PTB-resistant mutants of *P. atrosepticum* SCRI1043 and *P. brasiliense* 1692 emerged from the zone of inhibition and were selected. Subsequently, genomic DNA from *P. atrosepticum* SCRI1043 wild-type, *P. atrosepticum* SCRI1043 PTB-resistant mutant, *P. brasiliense* 1692 wild-type, *P. brasiliense* 1692 PTB-resistant mutant A and *P. brasiliense* 1692 PTB-resistant mutant B were isolated using a previously described method [19]. DNA quantity was assessed using a NanoDrop 1000 spectrophotometer (Thermo Fisher Scientific) and a Qubit 3.0 fluorometer (Thermo Fisher Scientific), and DNA purity was assessed using the Agilent 2100 BioAnalyzer system (Agilent). DNA libraries were prepared by the Cornell Genomics Facility (Cornell University, USA) using the Nextera DNA Flex library prep kit (Illumina), before whole-genome sequencing on an Illumina MiSeq with the 500 bp kit. Raw reads were processed to remove adaptor and low-quality sequences using Trimmomatic v0.39 [20]. Sequencing reads were aligned to the *P. atrosepticum* SCRI1043 genome [National Center for Biotechnology Information (NCBI) accession no. NC_004547.2] and *P. brasiliense* 1692 genome (NCBI accession no. CP047495) using bwa v0.7.17 [21]. Genome sequences of spontaneous resistant mutant strains were aligned with the genome sequence of the WT to identify mutations. Following alignments, differences were determined between the WT and the PTB mutant strains using BreSeq v0.32.1 with default settings [22]. Mutations were verified by PCR amplification and Sanger sequencing of the genomic regions.

### Swimming assays

*Dickeya* and *Pectobacterium* spp. were grown overnight on LB agar. The swimming medium consisted of LB with an agar concentration of 0.3 % (w/v). The medium was allowed to solidify for 3 h, then bacterial motility was assessed by stabbing the swimming medium with an inoculating needle with bacteria cells from the LB agar plates and measuring the diameter of the outer edge after 48 h at room temperature. The assay was performed in triplicate and repeated three times. A two-tailed Student's *t*-test (unequal variances) was used to check the statistical significance of differences between treatments (*P* value <0.05). The vertical lines in the graphs represent the standard deviation of the mean.

### Virulence assays and the effect of PTB spray

The potato stem virulence assay was adapted from a previously described method [23]. One-month-old potato plants were wounded 10 cm above the soil level by stabbing with a sterile 200 µl pipette tip halfway into the stem at an angle of 45 degrees. The wounding site was left to dry for 5 min before inoculating the potato stems with 10 µl of 2×10^9^ c.f.u. ml^−1^ (OD_600_ ≈ 2.0) overnight culture of *P. atrosepticum* SCRI1043 WT or its PTB-resistant mutant strain, previously washed and re-suspended in sterile 10 mM MgSO_4_. The wounding sites inoculated with bacteria were sprayed with 20 mM PTB (treatment) or water (control) at 24 h post-inoculation. Lesion lengths were measured at 4 days post-inoculation from six plants per treatment. Each experiment was repeated three times. A Student’s two-tailed *t*-test was used to determine the statistical significance of differences between the lesion lengths on PTB- and water-treated plants, as well as between the lesion lengths caused by the WT and the PTB-resistant mutant. The vertical lines in graphs represent the standard deviation of the mean.

### Complementation of the *P. atrosepticum* and *P. brasiliense* PTB-resistant mutants

PCRs were performed using the primer pairs shown in Table 1. The complementation constructs each contained 11–65 bp upstream of the target gene and the complete ORFs of the target genes, which were cloned into pML122 [24] at its *Bam*HI site. Each PCR contained the following: in 100 µl total reaction volume, 65 µl water, 20 µl 5× Q5 reaction buffer, 2 µl 10 mM dNTPs, 5 µl each primer at 10 µM, 1 µl Q5 high fidelity polymerase (New England Biolabs) and 2 µl genomic DNA at 50 ng µl^−1^. Cycling conditions were: 98 °C for 30 s; 40 cycles of 98 °C for 10 s, 72 °C (*cpdB, prfB, prmC*) or 67 °C (*supK*) for 30 s, 72 °C for 60 s; followed by a final extension of 72 °C for 2 min; with a 4 °C hold. PCRs were cleaned (Monarch PCR and DNA cleanup kit; New England Biolabs), and both the insert and the pML122 vector were digested with *Bam*HI-HF (New England Biolabs). The DNA insert and vector were ligated using T4 DNA ligase (New England Biolabs) and were used to chemically transform *Escherichia coli* TOP10 cells (Invitrogen). Plasmids were isolated using the Monarch plasmid miniprep kit (New England Biolabs). The inserted fragments were sequenced (Table S1, available with the online version of this article) at the Cornell Biotechnology Resource Center (BRC) to confirm that the cloned sequences and their relative position to the *nptII* promoter were correct. Each plasmid was introduced into electrocompetent *P. atrosepticum* SCRI1043 or *P. brasiliense* 1692 strains via electroporation and plated on LB agar with 10 µg gentamicin ml^−1^ and 50 µg kanamycin ml^−1^ for selection.

**Table 1.** Primers used for PCR in experiments complementing *P. atrosepticum* SCRI1043 and *P. brasiliense* 1692 PTB-resistant mutants (fwd, forward primer; rev, reverse primer)

Organism	Target gene	Primer sequence	Size (bp)
* P. atrosepticum* SCRI1043	*cpdB*	Fwd 5′-ggtggt**ggatcc**CCCCGATCGCCTCACGCG-3′	2035
		Rev 5′-ggtggt**ggatcc**GCGTTAAGCGCTTATTT-3′	
* P. atrosepticum* SCRI1043	*supK*	Fwd 5*′*-ggtggt**ggatcc**CCGCTAGAATGGCGGGTTAC*-*3*′*	1197
		Rev 5*′*-ggtggt**ggatCC**ATGCGAATTCTTATAA*-*3*′*	
* P. brasiliense* 1692	*prfB* (*supK*)	Fwd 5*′*-ggtggt**ggatcc**AAAACCGCATTCAGGATC*-*3*′*	1119
		Rev 5*′*-ggtggt**ggatcc**ATTCAGCCATGTGAATTC*-*3*′*	
* P. brasiliense* 1692	*prmC*	Fwd 5*′*-ggtggt**ggatcC**TGATTCAACCGGTTGTGCAG*-*3′	954
		Rev 5′-ggtggt**ggatcc**AGTTATCACGTCCATTGCTC-3′	

## Results

### Antibacterial activity of PTB on *Dickeya* and *Pectobacterium* spp.

**Fig. 1.**
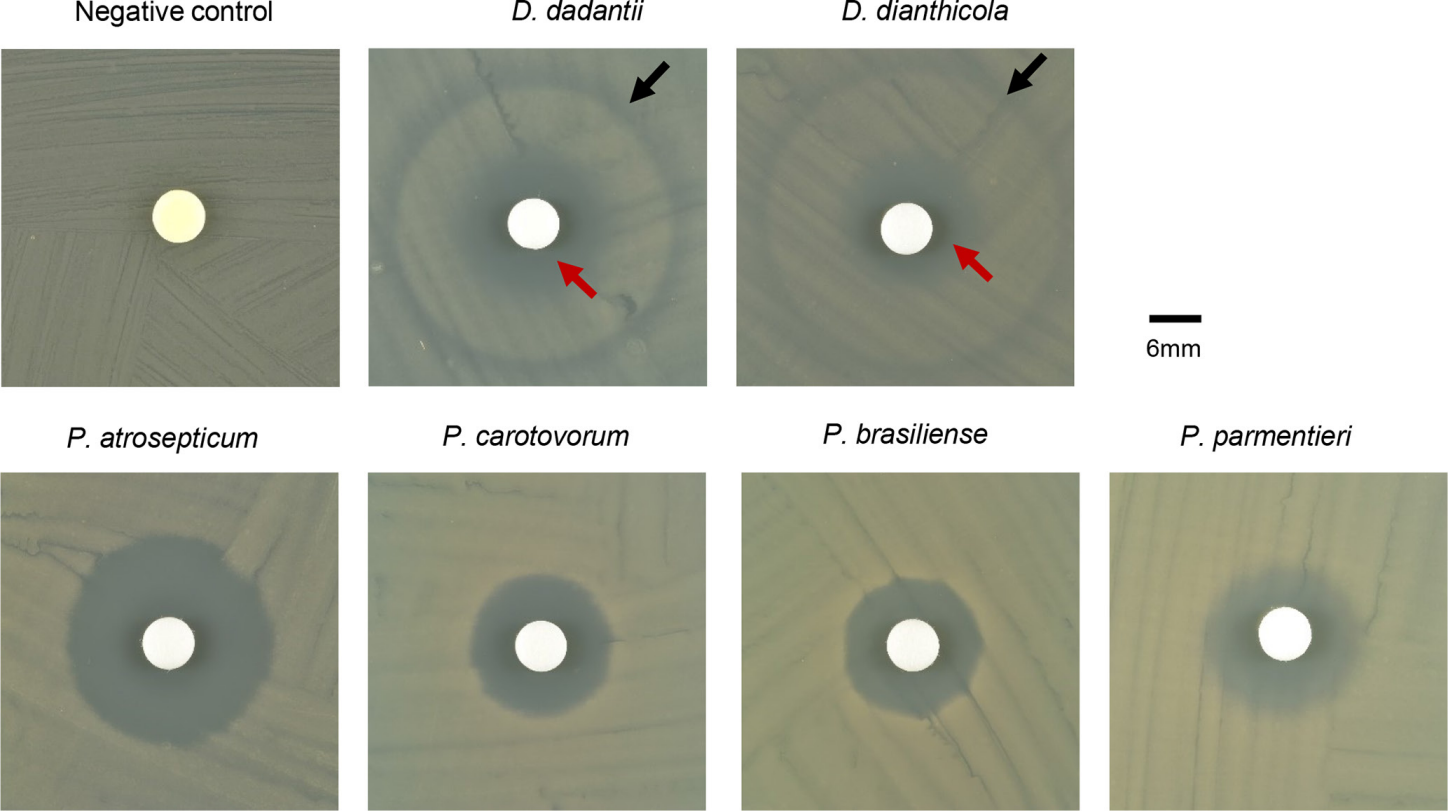
The disc diffusion assay of antibacterial activity of PTB on *Dickeya* and *Pectobacterium* spp. Experiments were repeated at least three times and representative photos show the zone of inhibition of each bacterium. The black arrows point to the outer peripheral zone and the red arrows to the inner peripheral zone.

**Fig. 2.**
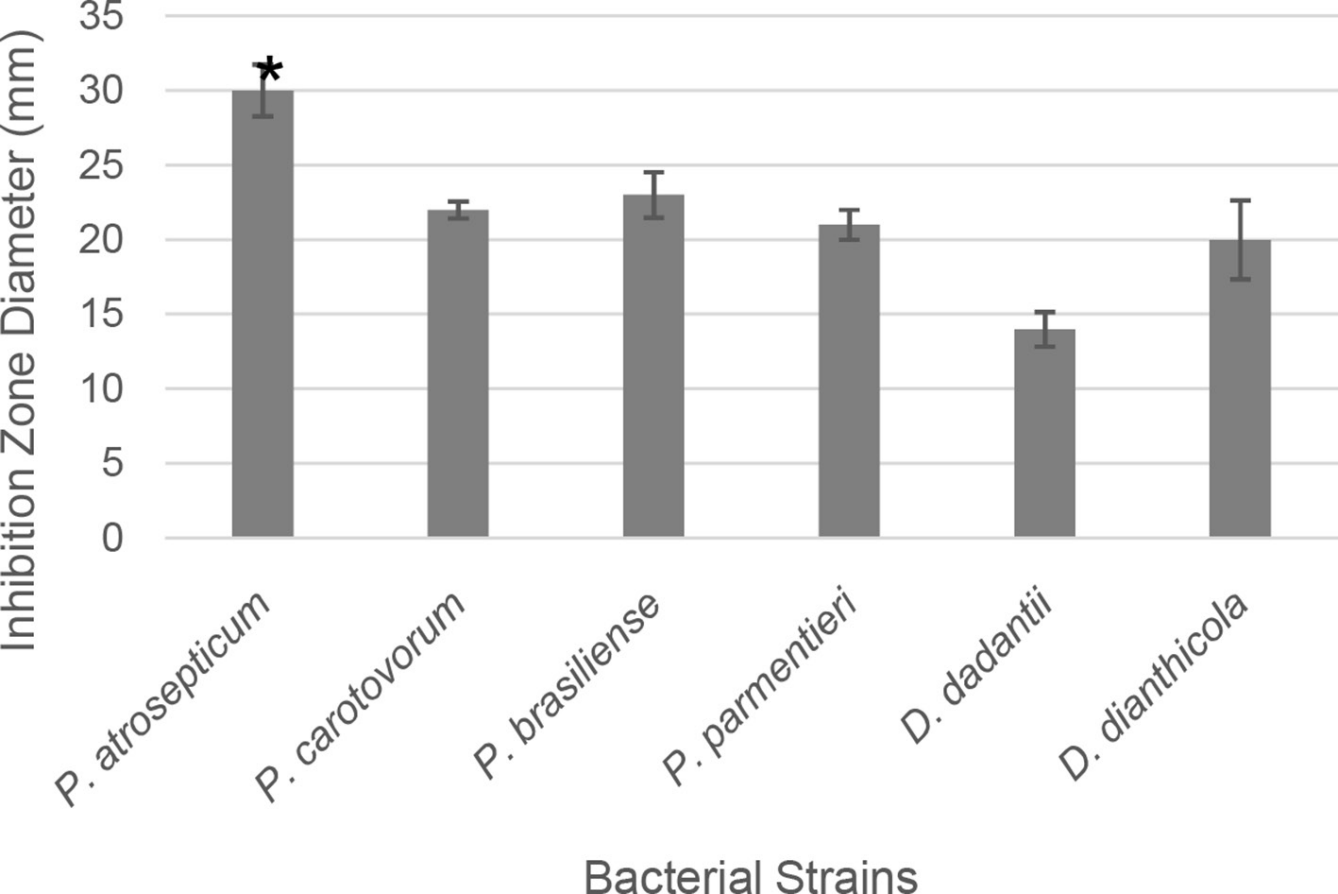
Diameters of the bacterial growth inhibition zones resulting from the PTB disc diffusion assay. The experiments were done in triplicate and repeated at least three times. The vertical lines represent the standard deviation of the mean.

Two *Dickeya* species and four *Pectobacterium* species were tested for their sensitivity to PTB using the disc diffusion method. The effect of 20 µl of 500 mM PTB on bacteria revealed that PTB was effective in suppressing the growth of *P. atrosepticum* SCRI1043, *P. carotovorum* WPP14, *P. brasiliense* 1692 and *P. parmentieri* WPP163, as indicated by the round clearing zones surrounding the PTB discs (Fig. 1). In contrast to the inhibition zone observed with *Pectobacterium* spp., the inhibition zone with *D. dadantii* and *D. dianthicola* had two distinct clearing zones, as indicated by the arrows in Fig. 1. One zone was located in the outer peripheral region (black arrow) and another zone was in the inner peripheral region (red arrow). The diameters of the growth inhibition zones were measured (inner peripheral zone) (Fig. 2). The diameter of the growth inhibition zone was largest with *P. atrosepticum* SCRI1043 (*P* value <0.05), suggesting PTB was more effective on *P. atrosepticum* SCRI1043 compared to other bacterial strains (Fig. 2). Results with the negative control were as expected, with no inhibition zone around discs with water. Potassium chloride, dipotassium phosphate and potassium acetate with 1 M K^+^ did not show any antibacterial effects on *Dickeya* and *Pectobacterium* spp. (Fig. S1).

### Identification of PTB-resistant mutants of *P. atrosepticum* SCRI1043 and *P. brasiliense* 1692

**Fig. 3.**
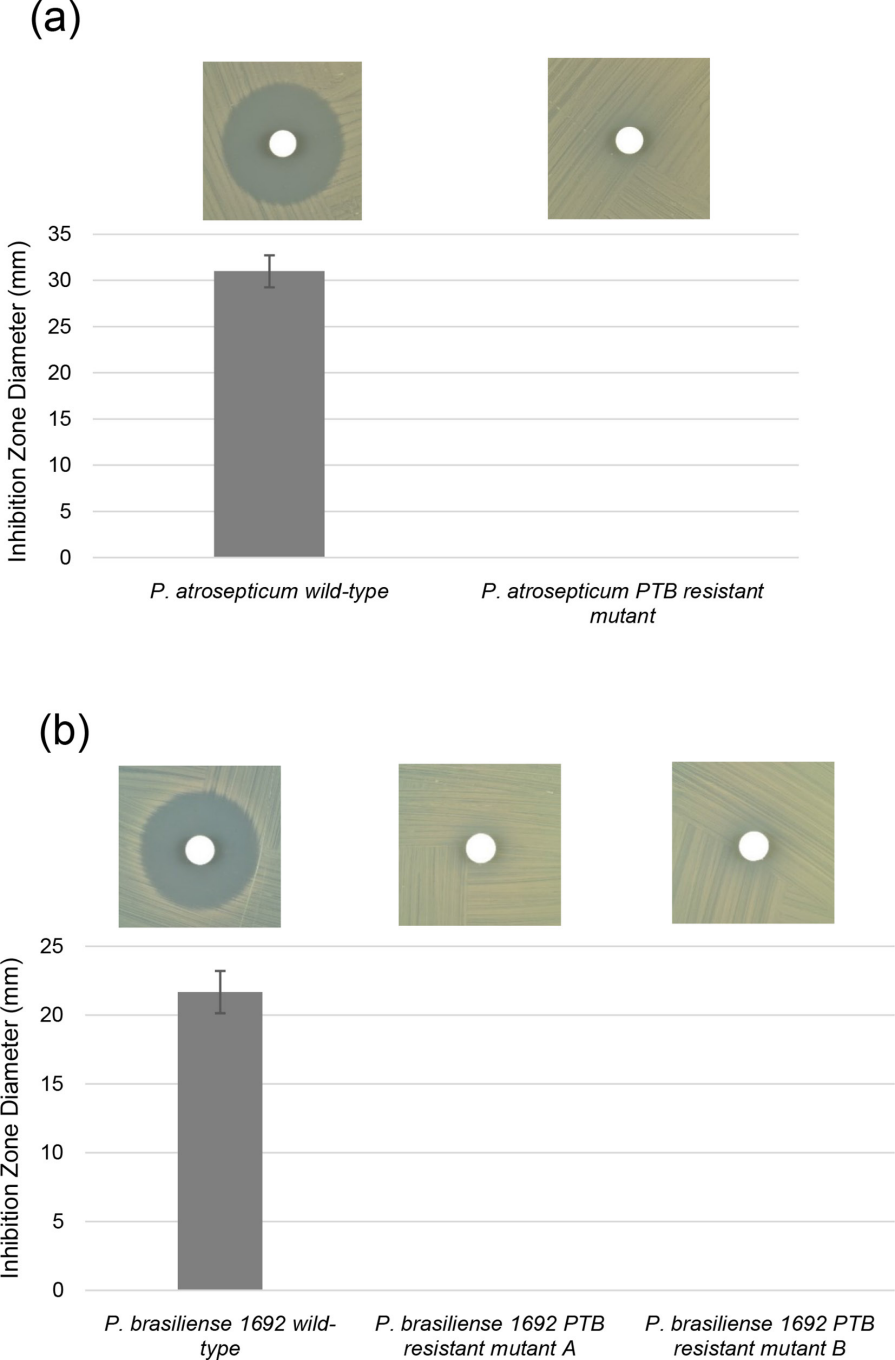
Diameters of the zones of inhibition resulting from PTB-impregnated discs for wild-type and PTB-resistant mutant(s). (a) *P. atrosepticum* SCRI1043 wild-type and the *P. atrosepticum* SCRI1043 PTB-resistant mutant. (b) *P. brasiliense* 1692 wild-type and *P. brasiliense* 1692 PTB-resistant mutant A and mutant B. The experiments were repeated at least three times and representative pictures show the zone of inhibition of each bacterium. The vertical lines represent the standard deviation of the mean.

**Fig. 4.**
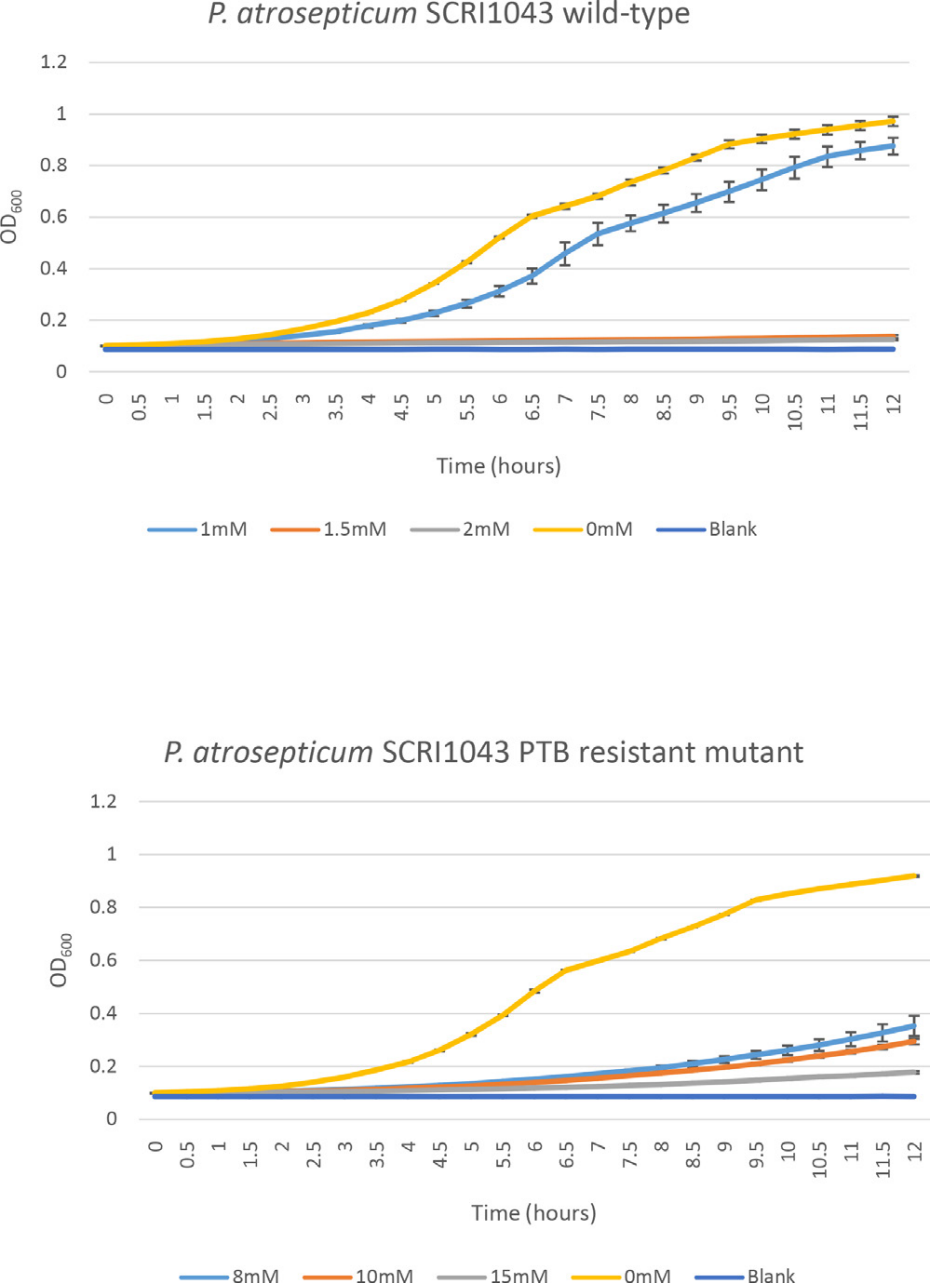
Growth (OD_600_) was monitored over a 12 h period for *P. atrosepticum* wild-type and the *P. atrosepticum* PTB-resistant mutant. Each treatment had four replicates and each experiment was repeated at least three times. One representative graph is shown. The vertical lines represent the standard deviation of the mean.

**Fig. 5.**
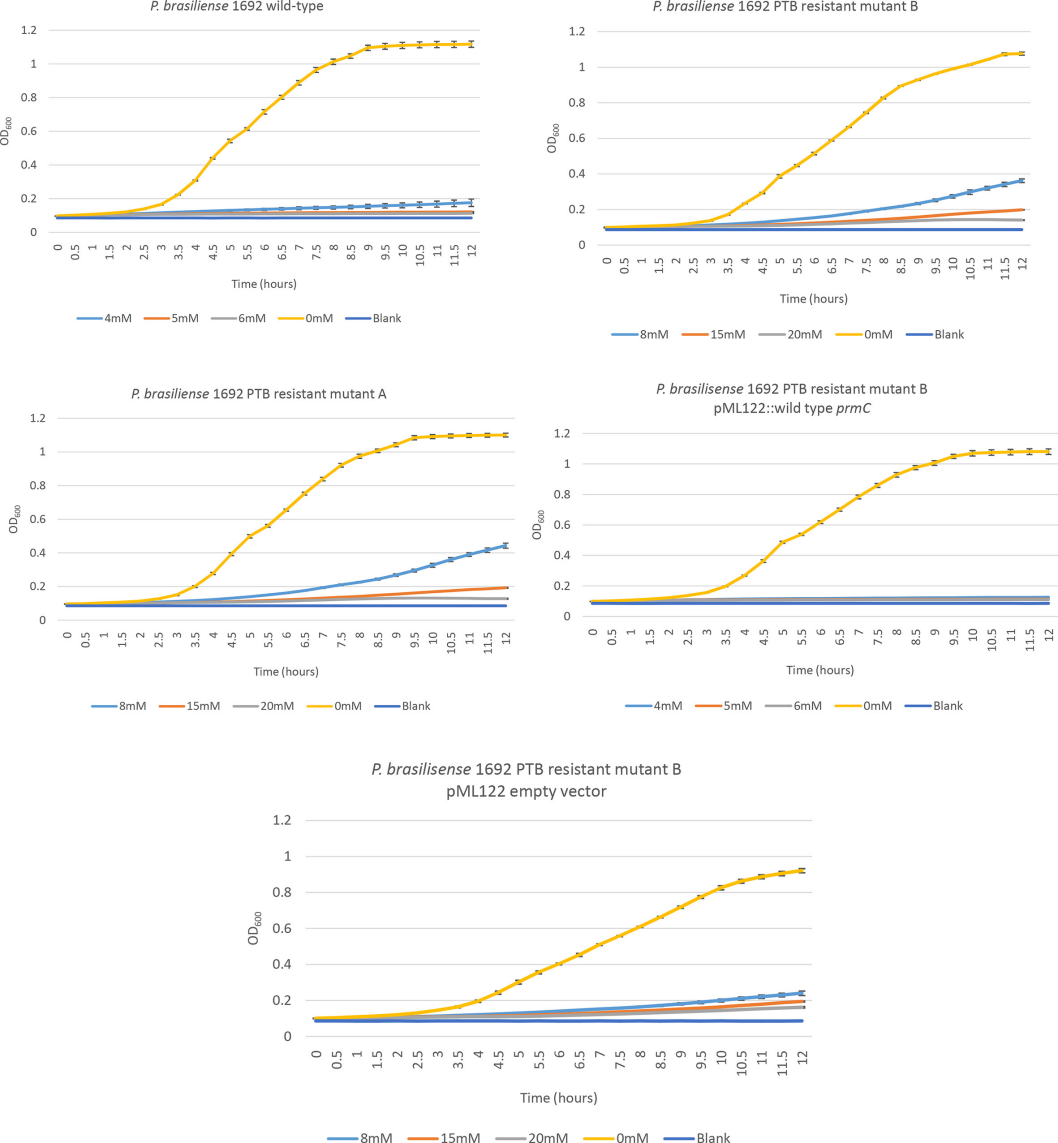
Growth (OD_600_) was monitored over a 12 h period for *P. brasiliense* wild-type and the *P. brasiliense* PTB-resistant mutant A, *P. brasiliense* PTB-resistant mutant B, *P. brasiliense* 1692 PTB-resistant mutant B with pML122::wild-type *prmC* and *P. brasiliense* PTB-resistant mutant B with pML122 empty vector. Each treatment had four replicates and each experiment was repeated at least three times. One representative graph is shown. The vertical lines represent the standard deviation of the mean.

One spontaneous *P. atrosepticum* SCRI1043 PTB-resistant mutant and two spontaneous *P. brasiliense* 1692 PTB-resistant mutants appeared in the inhibition zones on agar plates at 3 days post-incubation. These PTB-resistant mutants were re-streaked onto fresh LB agar plates and species were confirmed to be *P. atrosepticum* SCRI1043 or *P. brasiliense* 1692 by PCR amplification and Sanger sequencing of the *dnaX* gene (data not shown). Upon retesting, growth of the PTB-resistant mutants was no longer inhibited by PTB after 24 h compared to the respective wild-type strains (Fig. 3a, b). No growth defect of the PTB-resistant mutants was observed when they were grown in LB medium when PTB was not present, as shown by the growth curves in Figs 4 and 5. For WT *P. atrosepticum* SCRI1043 and its PTB-resistant mutant, the PTB concentrations at which no visible growth was detected after 12 h incubation were 1.5 and 15 mM, respectively (Fig. 4). For WT *P. brasiliense* 1692 and its PTB-resistant mutant A and mutant B, the PTB concentrations at which no visible growth was detected after 12 h incubation were 5, 20 and 20 mM, respectively (Fig. 5).

### Mutations found in *P. atrosepticum* SCRI1043 and *P. brasiliense* 1692 PTB-resistant mutants

To understand the mode of action of PTB on *Pectobacterium *, genomic DNA of *Pectobacterium* WT strains and PTB-resistant strains were subjected to Illumina sequencing to determine whether there were genomic differences. Both *P. atrosepticum* SCRI1043 WT and the PTB-resistant mutant sequences were compared to the reference genome. One point mutation in the *hisS* gene was found in both strains compared to the reference genome at the position 3 602 124, with a nucleotide change from A to T. This results in an amino acid change from phenylalanine to isoleucine. Since this mutation was found in the genomic sequence of our *P. atrosepticum* SCRI1043 wild-type strain as well as the PTB-resistant mutant compared to the reference genome, this mutation is not likely to be involved in PTB resistance and, therefore, was not investigated further. Additionally, one of the point mutations in *P. brasiliense* 1692 PTB-resistant mutant B compared to WT at the position 1 041 266, with a nucleotide change from G to A, resulted in a silent mutation, which was not investigated further.

**Fig. 6.**
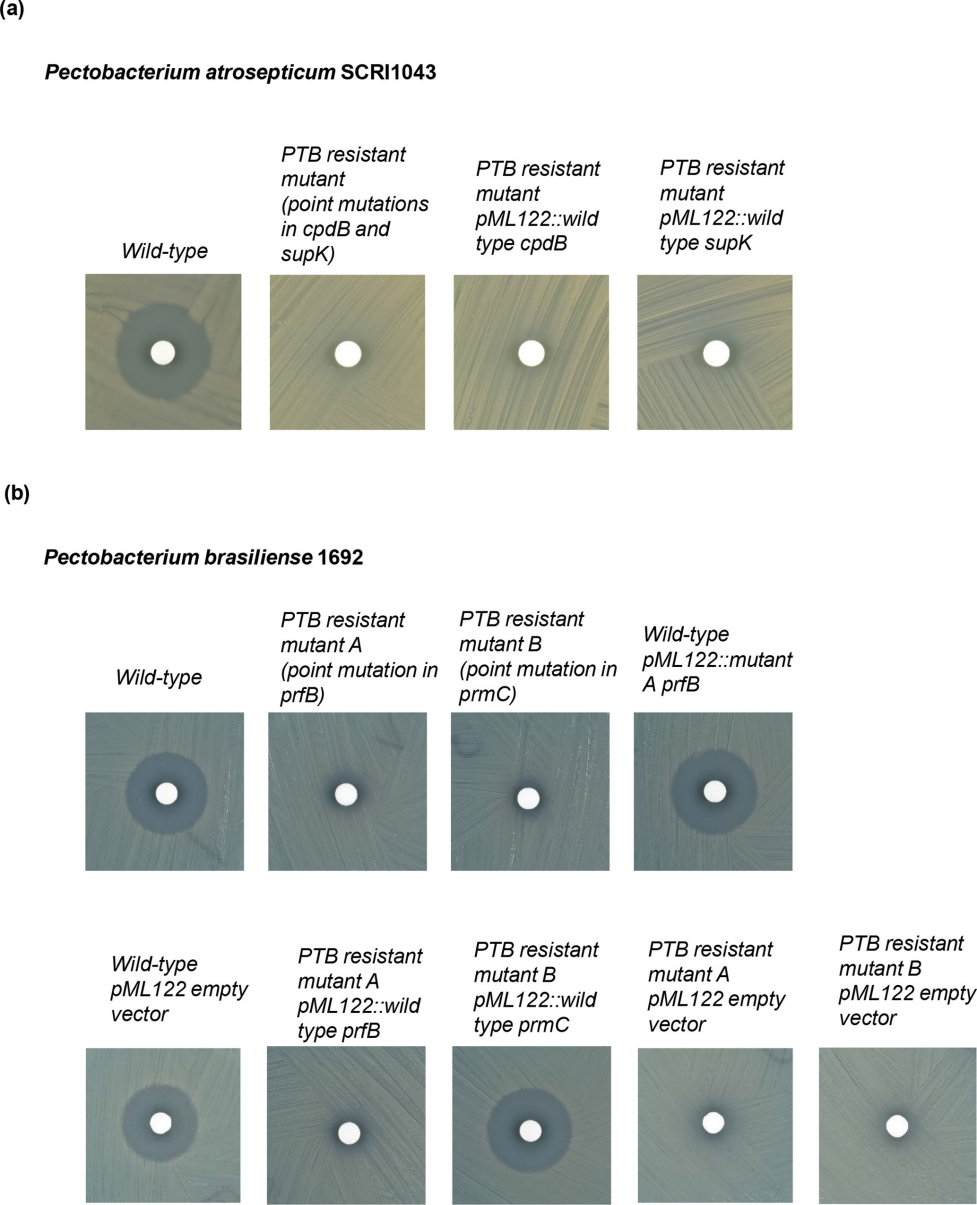
The disc diffusion assay testing bacterial sensitivity to PTB. (a) *P. atrosepticum* SCRI1043 wild-type, mutant strain and complementation strains. (b) *P. brasiliense* 1692 wild-type, mutant strains and complementation strains. The experiment was repeated at least three times and representative photos show the zone of inhibition of each bacterium.

Two point mutations in the genome sequence were found in the PTB-resistant mutant compared to WT *P. atrosepticum* SCRI1043. One point mutation was located in the gene *cpdB* with a nucleotide change from G to A and an amino acid change from valine to methionine. *cpdB* encodes a 2',3'-cyclic-nucleotide 2'-phosphodiesterase. The other point mutation was located in the gene *supK*, resulting in a nucleotide change from C to T and an amino acid change from arginine to cysteine (Table 2). *supK* encodes a putative peptide chain release factor 2. Protein structures predicted by Phyre2 [25] indicated different folding structures of CpdB and SupK resulted from the point mutations (Fig. S2). The mutations identified from Illumina sequencing and bioinformatics analysis were validated by amplifying the genes with PCR and Sanger sequencing (data not shown). The disc diffusion assay showed antibacterial activity of PTB on *P. atrosepticum* SCRI1043 wild-type, but no antibacterial activity on the *P. atrosepticum* SCRI1043 PTB-resistant mutant. To determine whether either the *cpdB* or the *supK* gene contributed to the PTB resistance, we cloned each of the genes individually and complemented the PTB-resistant mutant. The single complementation mutants of the *P. atrosepticum* PTB-resistant mutant still displayed the PTB-resistance phenotype (Fig. 6a). This suggests that both *cpdB* and *supK* might contribute to the observed phenotype.

**Table 2.** Details for the genes that harboured the mutations in the *P. atrosepticum* SCRI1043 PTB-resistant mutant and the two *P. brasiliense* 1692 PTB-resistant mutants

Gene name	Gene location	Mutated position	Mutation type	Mutation	Gene annotation
*cpdB* (in *P. atrosepticum* SCRI1043 PTB-resistant mutant)	4 051 288.4 053 240	4 052 221	Missense	G→A	2′,3′-Cyclic-nucleotide 2′-phosphodiesterase
*supK* (in *P. atrosepticum* SCRI1043 PTB-resistant mutant)	843 475.844 573	844 307	Missense	C→T	Putative peptide chain release factor 2
*prfB (supK*) (in *P. brasiliense* 1692 PTB-resistant mutant A)	842 107.843 205	842 469	Missense	C→T	Peptide chain release factor 2
*prmC* (in *P. brasiliense* 1692 PTB-resistant mutant B)	4 055 206.4 056 051	4 055 357	Missense	C→T	Peptide chain release factor N(5)-glutamine methyltransferase

One point mutation in the genome sequence was found in each of the PTB-resistant *P. brasiliense* 1692 mutants. In *P. brasiliense* 1692 PTB-resistant mutant A, the point mutation was located in the gene *prfB* (synonym *supK*), with a nucleotide change from C to T and an amino acid change from alanine to threonine. *prfB (supK*) encodes peptide chain release factor 2. In *P. brasiliense* 1692 PTB-resistant mutant B, the point mutation was located in the gene *prmC*, with a nucleotide change from C to T and an amino acid change from glycine to aspartic acid. *prmC* encodes peptide chain release factor N(5)-glutamine methyltransferase, which methylates the product encoded by *prfB (supK*) [26] (Table 2). The disc diffusion assay showed antibacterial activity of PTB against *P. brasiliense* 1692 WT, but no antibacterial activity against *P. brasiliense* 1692 PTB-resistant mutant A nor B.

To determine which gene was involved in PTB sensitivity, we cloned each of the genes of interest individually and complemented the respective PTB-resistant mutant [* P. brasiliense* 1692 PTB-resistant mutant A with pML122::wild-type *prfB (supK*) and *P. brasiliense* 1692 PTB-resistant mutant B with pML122::wild-type *prmC*]. The single complementation mutant of the *P. brasiliense* 1692 PTB-resistant mutant B with pML122::wild-type *prmC* restored the PTB sensitive phenotype (similar to wild-type), whereas *P. brasiliense* 1692 PTB-resistant mutant A with pML122::wild-type *prfB (supK*) still displayed the PTB-resistance phenotype (Fig. 6b). This suggests that in *P. brasiliense* 1692, *prmC* is required for sensitivity to PTB. For wild-type *P. brasiliense* 1692 and its PTB-resistant mutant A and mutant B, the PTB concentrations at which no visible growth was detected after 12 h incubation were 5, 20 and 20 mM, respectively (Fig. 5).

### Swimming motility of PTB-resistant mutants and complementation mutants compared to wild-type *Pectobacterium* spp.

**Fig. 7.**
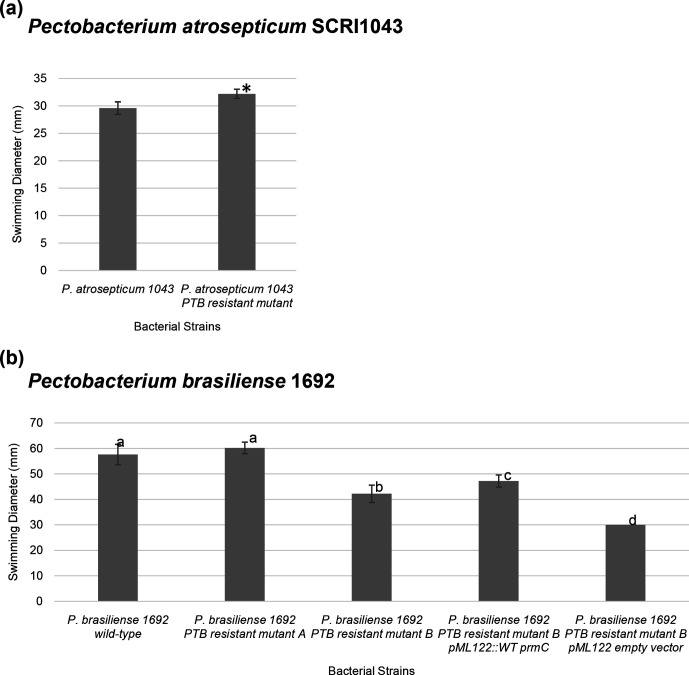
Diameters of the swimming zones of bacterial strains. The experiments were done in triplicate and repeated at least three times. (a) *P. atrosepticum* SCRI1043 wild-type and mutant strains, and their swimming diameters. (b) *P. brasiliense* 1692 wild-type, mutant strains and complementation strains, and their swimming diameters. The experiment was repeated at least three times. A two-tailed Student's *t*-test (unequal variances) was used to check the statistical significance of differences between treatments (*P value <0.05). The letters next to the bars in (b) depict significant differences between measurements. The vertical lines represent the standard deviation of the mean.

Motility plays an important role in bacterial virulence [27]. Additionally, bacterial cyclic dinucleotide phosphodiesterase can affect a number of bacterial phenotypes, such as motility, biofilm formation and virulence [28]. Therefore, the swimming motility of the PTB-resistant mutants, the complementation mutants and their corresponding wild-type strains were examined. In *P. atrosepticum* SCRI1043, the swimming motility of the PTB-resistant mutant was enhanced compared to wild-type *P. atrosepticum* SCRI1043 (*P*=0.003) on rich medium (Fig. 7a). However, complementation could not be restored (data not shown). For *P. brasiliense* 1692, the swimming motility of the PTB-resistant mutant B (mutation in *prmC*) was significantly reduced compared to wild-type *P. brasiliense* 1692 (*P*=0.0002). Complementation of the PTB-resistant mutant B with wild-type *prmC* gene partially restored the bacterial swimming motility (Fig. 7b).

### Efficacy of PTB when sprayed on the stems of potato inoculated with *P. atrosepticum*

**Fig. 8.**
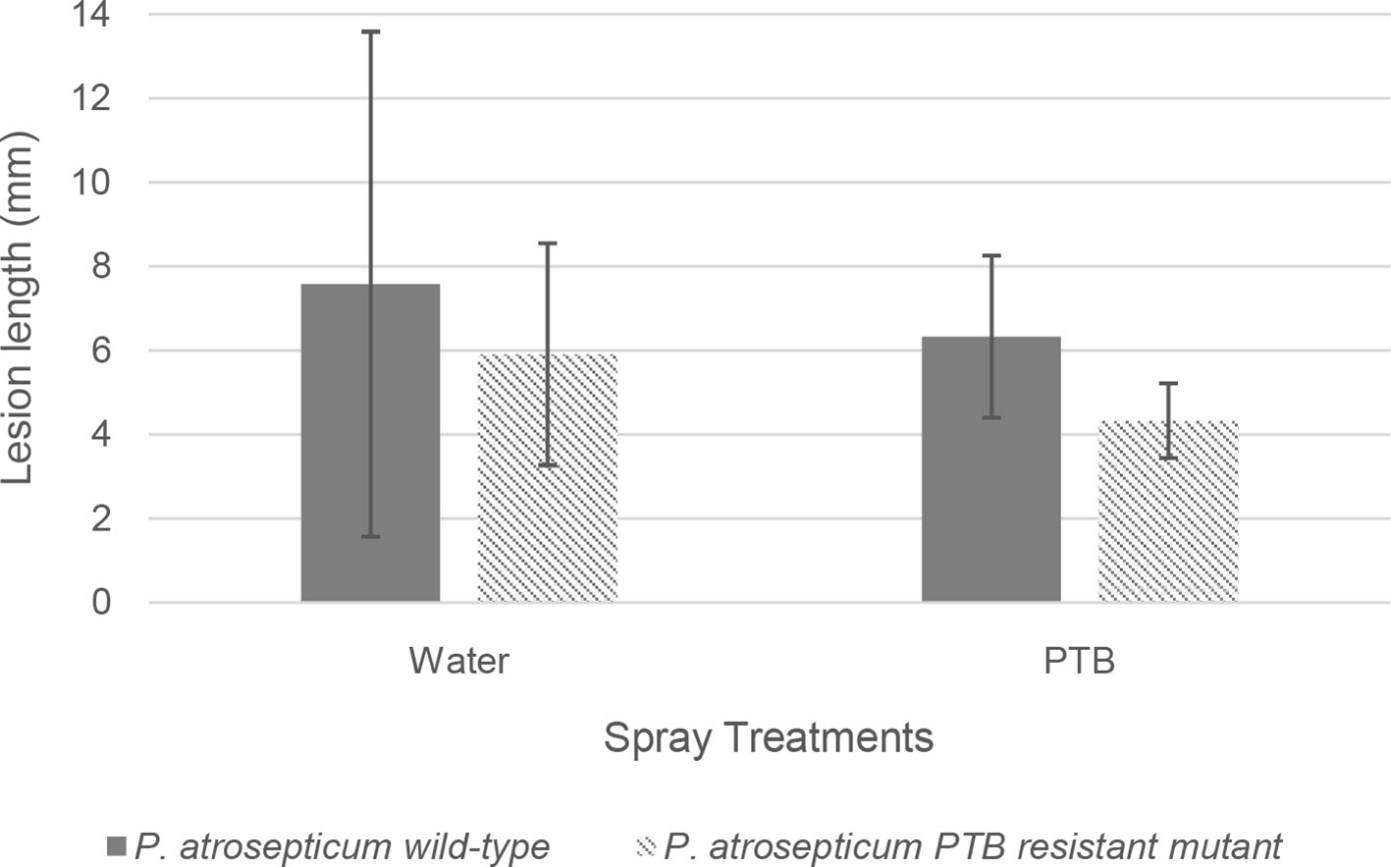
Lesion lengths on potato plants inoculated with *P. atrosepticum* wild-type or the PTB-resistant mutant, followed by spray treatment with water or 20 mM PTB at 24 h post-inoculation. Lesion lengths from six plants per treatment were calculated. Each experiment was repeated at least three times. One representative graph is presented. The vertical lines represent the standard deviation of the mean.

To evaluate the efficacy of PTB application post-* P *. *atrosepticum* infection, plant stems were inoculated with *P. atrosepticum *. Twenty-four hours after inoculation, potato plants were sprayed with 20 mM PTB solution or water. No phytotoxicity was observed on PTB-treated plants. There was no significant difference between the lesion lengths on stems when plants were treated with PTB post-bacterial challenge (Fig. 8). These results indicate that the application of PTB at 20 mM onto potato stems does not seem to reduce disease symptoms caused by *P. atrosepticum *. Although the *in planta* experiment did not demonstrate statistically significant differences between the lesion lengths for the WT compared to the mutant bacterial strain, nor between the water and PTB spray treatment, we observed shorter lesion lengths with *P. atrosepticum* inoculated stems treated with PTB compared to plants sprayed with water.

## Discussion

*Dickeya* and *Pectobacterium* displayed different sensitivity to PTB *in vitro* (Figs 1 and 2). *P. atrosepticum *, *P. carotovorum *, *P. brasiliense* and *P. parmentieri* displayed zones of inhibition as a result of the PTB-impregnated discs, with the largest inhibition effect observed in *P. atrosepticum *. The inhibition zone of *D. dadantii* and *D. dianthicola* showed two distinct clearing zones, one outer peripheral zone and one inner peripheral zone. This phenomenon has not been reported in *Dickeya* nor other bacterial species. We hypothesize that this pattern of clearing could be a result of either the development of resistance to PTB or phage. *Dickeya* spp. are known to harbour phages [29] and pro-phage like elements [30]. These elements could introduce novel traits to *Dickeya* spp. and alter fitness under stressful conditions [30].The six *Dickeya* and *Pectobacterium* spp. tested all showed sensitivity to PTB in LB, with inhibited growth over a period of time (data not shown). In the USA, *Dickeya* and *Pectobacterium* spp. are commonly found together on diseased potatoes in a disease complex [31]. Using chemical spray such as PTB to inhibit bacterial growth might be effective against *Pectobacterium *, but less effective against *Dickeya *.

PTB has been evaluated as an antifungal compound for control of post-harvest diseases [14, 15, 17, 32]. Potassium chloride, dipotassium phosphate and potassium acetate did not have inhibitory effects on *Dickeya* and *Pectobacterium* spp. (Fig. S1). This indicated that the antibacterial activity of PTB was not due to the effect of potassium alone. The inhibitory effects of boron have been observed on *E. coli* [33], *Staphylococcus aureus* [34] and '*Acinetobacter septicus'* [35]. Boron is essential for plant development and can improve plant resistance to disease, pest and environmental stresses. Boron, along with potassium and calcium, improves the quality of potatoes. Applications of boron foliar spray during potato plant growth have been shown to increase vitamin C concentration in the tubers, and decrease enzymatic discoloration and phenolic concentration in the tubers [36]. The long-term effects of applying PTB to control *Dickeya* and *Pectobacterium* are yet to be determined.

Several possible mechanisms for the inhibitory effect of borate have been proposed. It is known that boron-containing antibacterial agents are bacteriostatic, not bactericidal [33]. The binding of borate ions to chemical energy transporters such as ATP, NAD and NADH leads to impaired protein biosynthesis, disrupted mitochondrial function and halted cell division [37, 38]. Additionally, high salt concentrations disrupt osmolarity related processes and can reduce bacterial reproduction [39]. The *P. atrosepticum* PTB-resistant mutant harbours two point mutations within the genes of *cpdB* and *supK*. Moreover, the two *P. brasiliense* PTB-resistant mutants harbour point mutations within the genes of *prfB (supK*) and *prmC*. These findings provide insight into the mode of action and resistance mechanisms of PTB. Gene *cpdB* encodes the bifunctional periplasmic enzyme 2',3'-cyclic-nucleotide 2'-phosphodiesterase and is commonly found in Gram-negative bacteria [40, 41]. The *cpdB* mutant of *Yersinia enterocolitica* was unable to grow on 2',3'-cAMP as the only carbon source and displayed no significant change in virulence [42]. The *cpdB* mutant of *Salmonella enterica* increased the intracellular persistence in infected poultry [43]. Gene *supK/prfB* encodes the peptide chain release factor 2, which is a type of protein that participates in the stop-codon-dependent termination of polypeptide biosynthesis. Inactivation of *supK* can result in stop codon readthrough, growth retardation and induction of oxidative stress responses [44, 45]. Gene *prmC* encodes peptide chain release factor methyltransferase, which methylates peptide chain release factors. Release factor methylation is a conserved post-translational modification process in bacteria and is crucial to translation termination [26]. In *Pseudomonas aeruginosa, prmC* has been shown to be essential for motility, virulence and adaptation to anaerobic growth [46]. In *E. coli,* inactivation of *prmC* is lethal to cells in minimal medium and leads to a growth defect on rich media [44]. Since the mutation in *prmC* in *P. brasiliense* did not result in lethality, it suggests that *prmC* is not lethal in *P. brasiliense* or the mutation in *prmC* does not completely abolish all functions of PrmC. In *P. brasiliense,* complementation with *prmC* in the PTB-resistant mutant B, which harbours a mutated *prmC* gene, restored the PTB-sensitive phenotype (Fig. 6b). This indicated non-silent mutation(s) in *prmC* can lead to the PTB-resistant phenotype. Additionally, this PTB-resistant phenotype can be complemented when wild-type *prmC* gene is expressed *in trans*. Our discovery of mutations related to peptide chain release factors and their post-translational modification in different species of *Pectobacterium* supports important roles in the PTB resistance. We are currently constructing deletion mutants to clarify the roles of *cpdB* and *supK* in *P. atrosepticum *, as well as *prfB (supK*) and *prmC* in *P. brasiliense* resistance to PTB.

The swimming motilities of *Pectobacterium* PTB-resistant mutants were modified compared to their corresponding WT strains. Swimming motility is commonly considered a virulence trait of plant pathogenic bacteria. Altered swimming motility of the PTB-resistant strain not only can result in the reduced efficacy of using PTB as a chemical control, but also could result in selection for hyper-virulent strains in the long term [27]. We were unable to complement the phenotypes of *P. atrosepticum* SCRI1043 and *P. brasiliense* 1692 with *cpdB, supK* and *prfB (supK*). We hypothesize that *cpdB* is involved in numerous pathways, both *cpdB* and *supK* are needed to complement the phenotype, or higher levels of plasmid/gene expression are needed to complement the phenotype. We were not able to complement back *supK/prfB,* but were able to complement the gene (*prmC*) that methylates the product of *supK/prfB*. This might suggest a complex regulation that involves expression and/or methylation of the peptide release factor, which results in PTB sensitivity or resistance.

The efficacy of PTB application in controlling soft-rot disease of tomato fruit caused by *P. carotovorum* has been demonstrated previously [8]. Although our *in vivo* experiments did not demonstrate statistically significant differences between the lesion lengths of the wild-type compared to the mutant bacterial strain, nor between the water and PTB spray treatment, we did observe a trend of reduction of symptoms in *P. atrosepticum *-inoculated potato stems after spraying with PTB. (Fig. 8). Even though the pathosystem has inherent variability [47], further studies could include application of PTB at concentrations higher than 20 mM onto potato stems or application before bacterial inoculation to test reduction of disease symptoms caused by *P. atrosepticum *. Additionally, different plant parts (tubers, stems) could display different sensitivity to PTB treatment and need to be evaluated further. Further tests are needed with different PTB concentrations and different bacterial inocula to further investigate the efficacy of PTB application.

Understanding the mode of action of PTB would elucidate the factors contributing to the different sensitivities of *Dickeya* and *Pectobacterium* spp. and help evaluate the potential of using PTB as a chemical control for these species. Further investigations such as phytotoxicity, resistance development, effects on environments and humans will be needed to fully reveal the potential to control *Dickeya* and *Pectobacterium* spp. considering the chemical’s minimal impact to the environment and human health [48].

## Supplementary Data

Supplementary material 1

Supplementary material 2
