# Supplementary material for: Antibacterial activity and mode of action of potassium tetraborate tetrahydrate against soft-rot bacterial plant pathogens
Source: Microbiology (Reading). 2020 Jul 8;166(9):837–48. doi: 10.1099/mic.0.000948 (PMC7654739; doi:10.1099/mic.0.000948)
Supplement: Supplementary material 1 [file mic-166-837-s001.pdf]

Fig. S1. The disk diffusion assay showed no antibacterial activity of potassium choloride, dipotassium phosphate, or potassium acetate to *Dickeya* and *Pectobacterium* spp. The experiments were repeated at least three times and no zone of inhibition was observed.

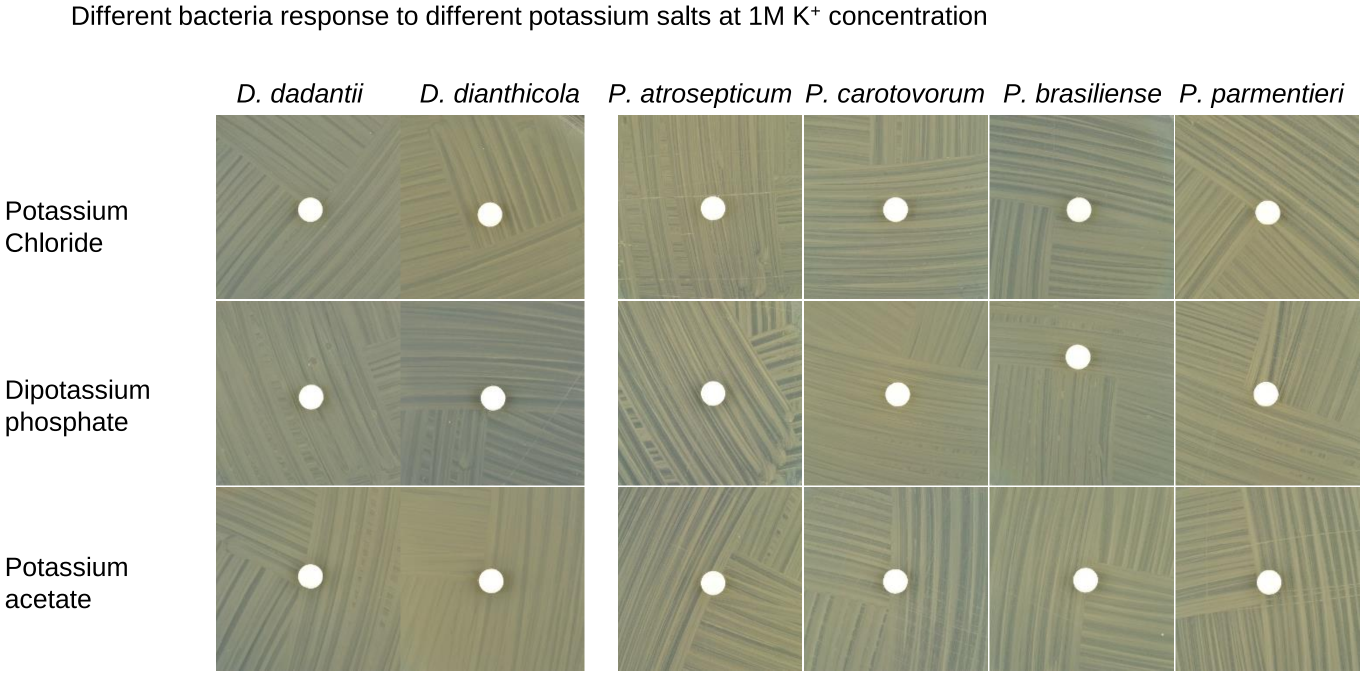

Fig. S2. Phyre 2 output of predicted protein structures of CpdB, SupK, PrfB, and PrmC comparing wild-type to the PTB resistant mutants.

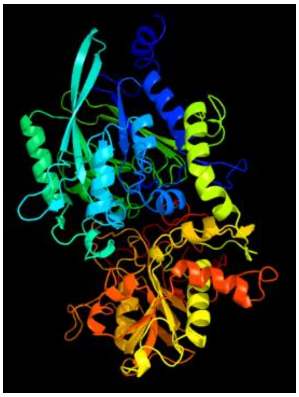

CpdB WT

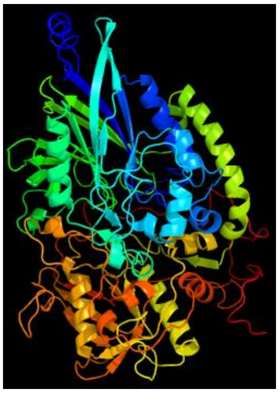

CpdB mutant

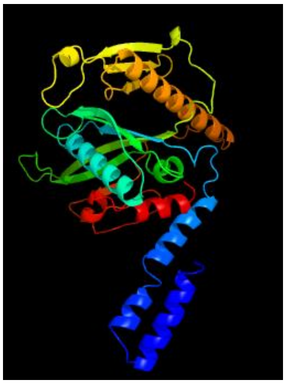

SupK WT

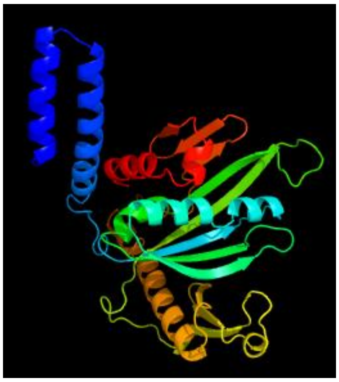

SupK mutant

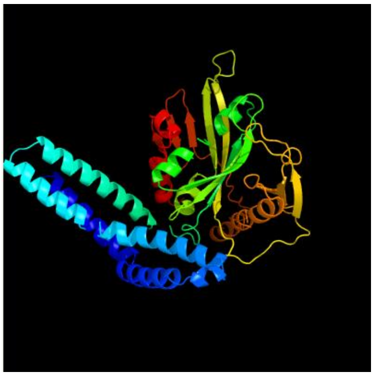

PrfB WT

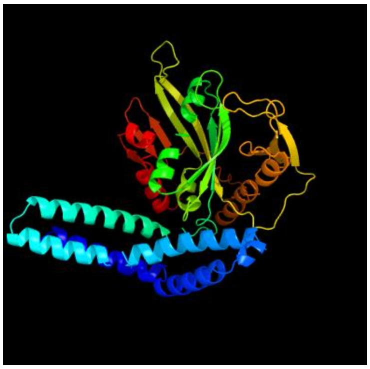

PrfB mutant

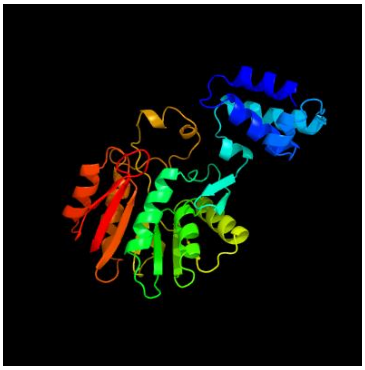

PrmC WT

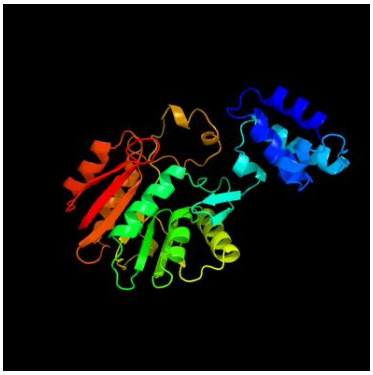

PrmC mutant
